# Mutational Analysis of *TYR*, *OCA2,* and *SLC45A2* Genes in Chinese Families with Oculocutaneous Albinism

**DOI:** 10.1002/mgg3.687

**Published:** 2019-06-14

**Authors:** Ye Lin, Xihui Chen, Ying Yang, Fengyu Che, Sijia Zhang, Lijuan Yuan, Yuanming Wu

**Affiliations:** ^1^ The General Hospital of the People's Liberation Army Beijing P.R. China; ^2^ Department of Biochemistry and Molecular Biology Center for DNA Typing, Air Force Medical University Xi'an Shaanxi P.R. China; ^3^ Shaanxi Institute of Pediatric Diseases, Xi’an Children’s Hospital Xi’an Shaanxi P.R. China; ^4^ Department of General Surgery Tangdu Hospital, Air Force Medical University Xi’an Shaanxi P.R. China

**Keywords:** *OCA2*, Oculocutaneous albinism, prenatal diagnosis, *SLC45A2*, *TYR*

## Abstract

**Background:**

Oculocutaneous albinism (OCA) is a group of heterogeneous autosomal recessive genetic disorder of melanin synthesis results in hypopigmented hair, skin, and eyes. OCA type 1, OCA type 2, and OCA type 4, which are respectively caused by mutations in *TYR*, *OCA2,* and *SLC45A2* have high morbidity rates in Asia.

**Methods:**

*TYR*, *OCA2,* and *SLC45A2* mutation analysis was carried out on 18 nonconsanguineous OCA patients and four fetuses were included for prenatal diagnose. Three genes of all individuals were amplified by polymerase chain reaction and examined by Sanger sequencing. The pathogenicity of the detected mutations were analyzed by Mutation Taster, PolyPhen 2, and SIFT software, and the conservation of the substituted amino acids were analyzed by MEGA software.

**Results:**

Eleven *TYR* mutations, three *OCA2* mutations, and two *SLC45A2* mutations were identified in 14 OCA type 1 patients, two OCA type 2 patients, and two OCA type 4 patients. c.1021A>G, p.R341G in *TYR*, c.1096_1104del, p.V366*, and c.1079C>T, p.S360F in *OCA2* were novel. One of the four fetuses carried compound heterozygous mutation of *TYR* and became spontaneous abortion, the other three carried no mutations and appeared normal at birth.

**Conclusion:**

In this study, specific clinical characteristics of OCA patients were described. Three novel pathogenic mutations were identified which will enrich the mutation spectrum of OCA, and the prenatal genetic screening in fetus at risk of OCA can provide vital information for genetic counseling.

## 1 INTRODUCTION

Albinism is a heterogeneous genetic disorder of melanin synthesis. According to the patients’ pathological changes sites, it can be divided into ocular albinism (OA) and oculocutaneous albinism (OCA). OA patients have only hypopigmented eyes while OCA patients have a more widely affected area including hair, skin, and also associated with photophobia, nystagmus, strabismus, and variably reduced visual acuity. Syndromic OCA caused by mutations in *HPS1, AP3B1, HPS3, HPS4, HPS5, HPS6, DTNBP1, BLOC1S3, PLDN, LYST, MYO5A, RAB27A*, or *MLPH* can also combine with severe immune deficiency or platelet storage pool deficiency (Kamaraj & Purohit, 2014). While mutations in the *TYR, OCA2, TYRP1, MATP (SLC45A2), SLC24A5,* or *C10ORF11* can cause OCA type 1, OCA type 2, OCA type 3, OCA type 4, OCA type 6, and OCA type 7, respectively (Kamaraj et al., 2014), which are identified as nonsyndromic OCA.

Nonsyndromic OCA is a group of heterogeneous autosomal recessive disorders that affect one in 20,000 individuals worldwide, while the prevalence of the different subtypes of albinism varies considerably among the different ethnic backgrounds (Hutton & Spritz, 2008a; Rooryck et al., 2008). OCA type 1 is caused by homozygous or compound heterozygous mutations in *TYR* (OMIM #606933) located on chromosome 11q14. The protein it encodes is called tyrosinase, which is a copper‐containing enzyme catalyzing the first two steps in melanin biosynthesis pathway required for melanin biosynthesis in the melanocytes of hair follicles, skin, and eyes (Cooksey et al., 1997). OCA type 1 is the most common subtype founded in Caucasians and accounts for about 50% of OCA cases worldwide (Hutton & Spritz, 2008b). OCA type 2 also called brown OCA, is caused by mutations in *OCA2* (also known as *P* gene, OMIM #611409) encoding a melanosome‐specific transmembrane protein called P protein, which is required for melanin production by mediating a chloride‐selective anion conductance (Bellono, Escobar, Lefkovith, Marks, & Oancea, 2014; Kamaraj et al., 2014; Passmore, Kaesmann‐Kellner, & Weber, 1999). This type is most common in Africa and accounts for 30% of OCA cases worldwide (Okoro, 1975; Puri et al., 1997). OCA type 3, also known as rufous OCA, is mostly caused by the mutations in *TYRP1* (OMIM #115,501). This gene encodes tyrosinase related protein 1 (Tyrp1) which is involved in the maintenance of melanosome structure and affects melanocyte proliferation and cell death (Fang, Tsuji, & Setaluri, 2002; Hirobe & Abe, 1999; Kobayashi, Imokawa, Bennett, & Hearing, 1998). Type 3 is hardly ever seen in Caucasians but affects almost 1/8,500 individuals in southern Africa and 3% of OCA cases worldwide (Rooryck et al., 2008). OCA type 4 is caused by mutations in the membrane‐associated transporter protein gene (SLC45A2, previously known as MATP, OMIM #606202). The SLC45A2 protein expresses in melanocyte cell lines and plays a role as melanosome membrane‐associated transporter (Fukamachi, Shimada, & Shima, 2001; Harada, Li, El‐Gamil, Rosenberg, & Robbins, 2001). This type accounts for 17% of OCA cases worldwide and 25% in Japan (Inagaki et al., 2004; Rooryck et al., 2008). OCA type 5, type 6, and type 7 are three recently identified types of nonsyndromic OCA. Type 5 has been mapped to the human chromosome 4q24, which is a novel locus for nonsyndromic OCA (Kamaraj et al., 2014; Visser, Kayser, Grosveld, & Palstra, 2014), and type 6 and type 7 are respectively caused by mutations in *SLC24A5* and *C10orf11* (Wei et al., 2013). In Chinese Han population, comprehensive molecular analysis has revealed the spectral distribution of Chinese OCA: the three commonest types are OCA type 1, OCA type 2, and OCA type 4 with the prevalence of 64.3%, 11.7%, and 15.6%, respectively (Wei, Zang, Zhang, Yang, & Li, 2015).

Further research of albinism associated genes can help to develop molecular tools used for prenatal diagnosis of albinism. By far, prenatal screening of albinism by molecular analysis has been performed in Israeli families (Rosenmann et al., 2009), Taiwanese families (Hsieh et al., 2001), and Chinese families (Liu, Kong, Shi, Wu, & Jiang, 2014; Wei et al., 2015).

In this study, we performed mutational screening of *TYR*, *OCA2,* and *SLC45A2* in 18 Chinese OCA patients, two novel mutations in *OCA2* and one novel mutation in *TYR* were identified. Meanwhile, prenatal screening of *TYR* mutation was performed on four fetuses, which have provided important information for genetic counseling.

## 2 PATIENTS AND METHODS

### 2.1 Patients

Eighteen nonconsanguineous patients recruited from the DNA Genotype Center of the Air Force Medical University were clinically diagnosed as OCA according to the diagnostic criteria. Their symptoms included severely poor eyesight, refractive error and photophobia in eyes, and white or light‐yellow skin or hair which did not change with ages (normal pigmentation in a Chinese population is black hair, black eye, and yellow skin), obvious syndromic forms of OCA were ruled out because no abnormalities of the immune system or symptoms in other organs were found. *Written informed consent including genetic analysis and publication of personal photographs were obtained from the participators and this study was approved by the Medical Ethics Committee of the Air Force Military Medical University*. A total of 21 families were screened, including 3 families receiving prenatal genetic counseling. Three common OCA related genes including *TYR* (NC_000011.10), *OCA2* (NC_000015.10), and *SLC45A2* (NC_000005.10) were screened in the patients by Sanger sequencing. Given that OCA type 3 is seldom found in Asian patients, we did not test *TYRP1* in this study.

### 2.2 Gene sequencing

Peripheral blood samples were collected from the patients and their parents for genomic DNA isolation using a DNA extraction kit (RelaxGene Blood DNA System, Tiangen Biotech, Beijing, China).

PCR primers were designed by Primer Premier 5.0 containing the sequences of all 5 exons of *TYR* (Table S1), 25 exons of *OCA2* (Table S2), and 7 exons of *SLC45A2* (Table S3) including all coding exons and the exon/intronic flanking regions (Tables S1–S3).

The 50 μl PCR reaction mixture contained 50–100 ng template DNA, 25 μl 2 × Taq PCR MaterMix (Tiangen Biotech, Beijing, China), and 2 μl of each primer. PCR were carried out in the following steps: pre‐denatured at 95°C for 3 min followed by 35 cycles of denaturation at 95°C for 30 s, annealing at 50–60°C (according to different primers) for 30 s to 1 min, then extension at 72°C for 40 s, followed by the final extension at 72°C for 10 min. All PCR product sizes were verified by electrophoresing in 1.5% agarose gel at 100 V for 30 min and staining by ethidium bromide.

The expected sizes of the amplified PCR products were sequenced by Big Dye Primer Cycle Sequencing kit and the ABI 310 Genetic automated sequencer (Applied Biosystems, Foster city, CA, USA), according to the manufacturer's instructions.

The sequencing results were then compared with the reference sequences of the *TYR*, *OCA2,* and *SLC45A2* obtained from the Genome Bioinformatics Database of California Santa Cruz University and the suspected disease‐related mutations were then verified by family co‐segregation.

### 2.3 Prenatal testing

Three of the fetal DNA samples were obtained by amniocentesis performed at the 17th week of gestation, and one was isolated from aborted fetal tissues when the spontaneous abortion happened at the ninth week of gestation. Genomic DNA was extracted using a micro DNA extraction kit (TIANamp Micro DNA Kit, Tiangen Biotech, Beijing, China). Then the DNA was amplified and sequenced as described above.

### 2.4 In silico analysis of novel mutations

All the detected variations were assessed by the Albinism Databases (http://www.ifpcs.org/albinism/index.html), 1,000 Genomes Database (http://browser.1000genomes.org/index.html), gnomAD database (http://gnomad.broadinstitute.org/), and HGMD Professinal Database (http://portal.biobase-international.com/ghmd/pro/search_gene.php). The effects of novel mutations were further analyzed in silico. Mutation Taster (http://www.mutationtaster.org/), Polyphen‐2 (http://genetics.bwh.harvard.edu/pph2/), and SIFT (http://sift.jcvi.org/) were used to determine the pathogenicity of novel mutations. The protein stability changes upon novel mutations were assessed by I‐Mutant2.0 (http://folding.biofold.org/cgi-bin/i-mutant2.0.cgi). The conservative analysis of single amino acid site was achieved by MEGA software and the conformation changes of polypeptide caused by amino acid substitutions were simulated by SWISS‐MODEL (https://swissmodel.expasy.org).

## 3 RESULTS

**Figure 1:**
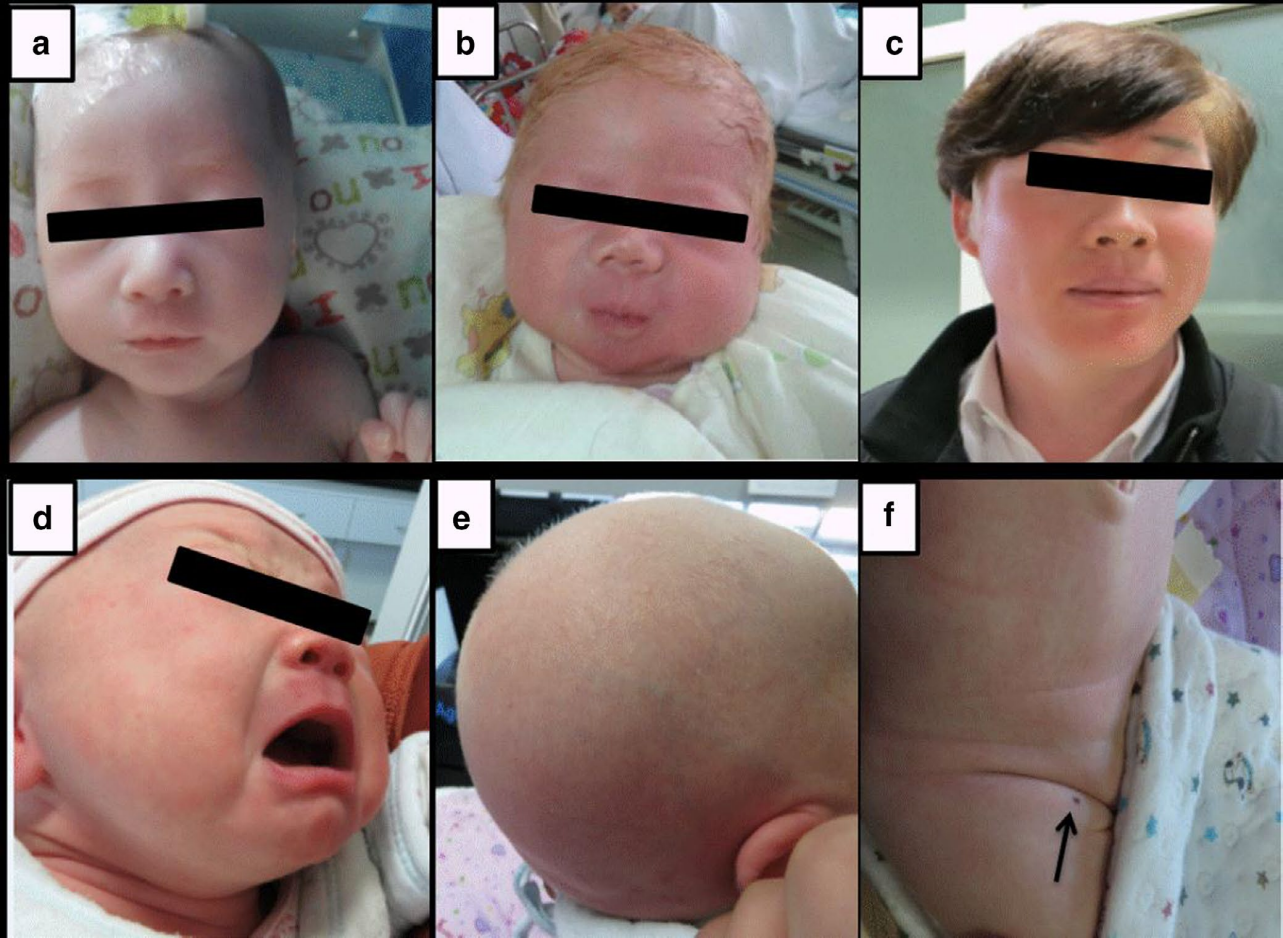
(a–d) Photographs of four patients with OCA; (e–f) Patient 15 with *OCA2* mutations has white skin but a pigmented nevus at groin (arrow)

In our study, 18 OCA patients were definitely diagnosed through genetic testing. Fourteen patients were diagnosed as OCA type 1 due to the *TYR* mutations, two patients were affected by *OCA2* mutations causing OCA type 2, another two patients were affected by *SLC45A2* mutations causing OCA type 4. All patients had typical OCA symptoms on their skin, hair, and iris. Clinical features and mutation alleles of these 18 patients are shown in Table 1. Interestingly, one of the patients (Patient 15 in Table 1) with *OCA2* mutations has white skin but a pigmented nevus at groin (Figure 1).

**Table 1:** Clinical characteristics and genotypes of the 18 patients in this study

Subject	Sex	Age (years)	Hair color	Skin color	Iris color	Molecular subtype	Causing gene	Exon	Subject genotype	
									Allele 1	Allele 2
1	F	0.13	White	White	Brown	OCA1	*TYR*	1/2	c.832C>T(p.R278*)	c.346C>T(p.R116*)
2*	M	40	White	White	Gray	OCA1	*TYR*	2	c.929_930insC(p.R311Lfs*7)	c.929_930insC(p.R311Lfs*7)
3	M	0.01	White	White	Unknown	OCA1	*TYR*	2	c.896G>A(p.R299H)	c.929_930insC(p.R311Lfs*7)
4*	M	0.01	White	White	Gray	OCA1	*TYR*	2	c.832C>T(p.R278*)	c.832C>T(p.R278*)
5	M	50	White	White	Red‐brown	OCA1	*TYR*	2	c.832C>T(p.R278*)	c.929_930insC(p.R311Lfs*7)
6	M	33	White	White	Gray	OCA1	*TYR*	2/3	c.896G>A(p.R299H)	c.1106A>G(p.Y369C)
7	M	3	Light‐yellow	White	Unknown	OCA1	*TYR*	2/4	c.1199G>T(p.W400L)	c.895C>T(p.R299C)
8	F	24	White	White	Gray	OCA1	*TYR*	2	c.929_930insC(p.R311Lfs*7)	**c.1021A>G(p.R341G)**
9	F	0.15	White	White	Gray	OCA1	*TYR*	2	c.896G>A(p.R299H)	c.929_930insC(p.R311Lfs*7)
10	M	28	White	White	Brown	OCA1	*TYR*	2	c.896G>A(p.R299H)	c.929_930insC(p.R311Lfs*7)
11	M	0.25	White	White	Unknown	OCA1	*TYR*	1	c.71G>A(p.C24Y)	c.636A>T(p.R212S)
12	F	28	White	White	Gray	OCA1	*TYR*	1/4	c.346C>T(p.R116*)	c.1199G>T(p.W400L)
13	F	26	White	White	Gray	OCA1	*TYR*	2	c.832C>T(p.R278*)	─
14	M	2	White	White	Gray	OCA1	*TYR*	2	c.929_930insC(p.R311Lfs*7)	─
15^#^	F	0.01	White	White	Unknown	OCA2	*P*	10	c.1096_1104del(p.V366*)	**c.1079C>T(p.S360F)**
16*	F	0.17	White	White	Brown	OCA2	*P*	8	c.833T>G(p.L278*)	c.833T>G(p.L278*)
17	F	0.06	White	White	Gray	OCA4	*SLC45A2*	1/2	c.328G>A(p.G110R)	c.478G>C(p.D160H)
18*	M	0.17	White	White	Gray	OCA4	*SLC45A2*	2	c.478G>C(p.D160H)	c.478G>C(p.D160H)

Note

F, female; M, male.

The asterisk (*) means this patient was in homozygous form.

The pound sign (#) indicates the patient has a pigmented nevus.

The dash (─) in the genotype column means a putative uncharacterized allelic mutation that may not be in *TYR* or *OCA2* and was not identified by the PCR primers used.

Novel mutations are in bold.

### 3.1 Mutations identification

**Figure 2:**
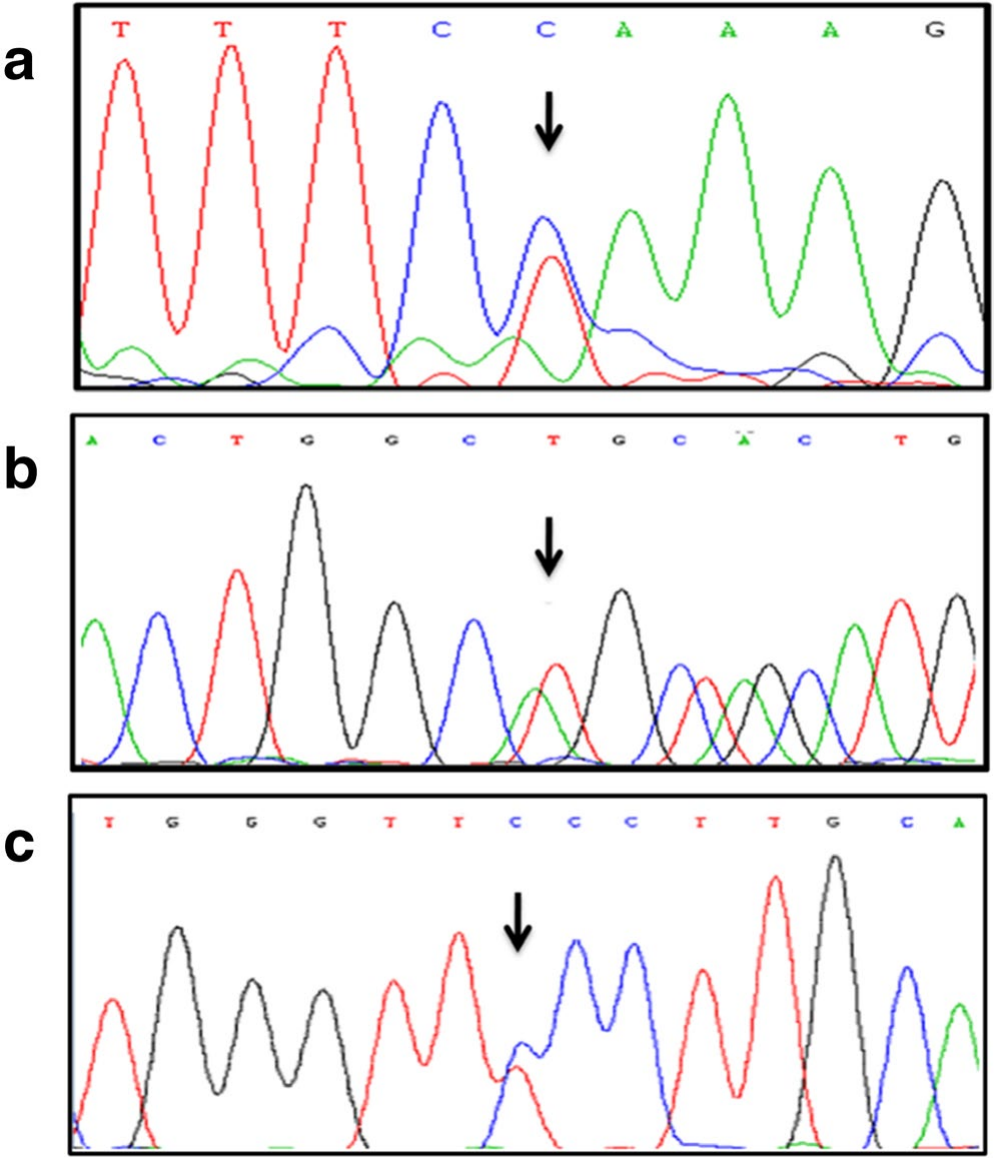
(a) c.1021A>G mutation in *TYR*; (b) c.1096_1104del mutation in *OCA2*; (c) c.1079C>T mutation in *OCA2*

Gene analysis revealed 10 different *TYR* mutation alleles, one novel missense mutation c.1021A>G (p.R341G) in *TYR* was identified (Figure 2a). Among the 14 patients who were affected by *TYR* mutation, ten subjects were compound heterozygous, two subjects were homozygous (Patient 2 and 4 in Table 1) and two subjects were single heterozygous of mutant *TYR* alleles (Patient 13 and 14 in Table 1). Three *TYR* mutations accounted for 60.7% of all the mutations in *TYR* in this study. The most common mutation was c.929_930insC(p.R311Lfs*7) (Exon 2) accounting for 8/28 of the *TYR* mutation alleles, followed by c.832C>T(p.R278*) (Exon 2) accounting for 5/28 and c.896G>A(p.R299H) (Exon 2) accounting for 4/28. All other identified mutations occurred in only one or two subjects (Table 2).

**Table 2:** OCA1 mutational alleles detected in this study

Gene	Mutation type	Nucleotide change	Amino acid change	Exon No.	Mutation frequency
*TYR*	Missense	**c.1021A>G**	**p.R341G**	Ex 2	1/28
		c.1106A>G	p.Y369C	Ex 3	1/28
		c.1199G>T	p.W400L	Ex 4	2/28
		c.636A>T	p.R212S	Ex 1	1/28
		c.71G>A	p.C24Y	Ex 1	1/28
		c.895C>T	p.R299C	Ex 2	1/28
		c.896G>A	p.R299H	Ex 2	4/28
	Nonsense	c.832C>T	p.R278*	Ex 2	5/28
		c.346C>T	p.R116*	Ex 1	2/28
	Insertation	c.929_930insC	p.R311Lfs*7	Ex 2	8/28
*OCA2*	Missense	**c.1079C>T**	**p.S360F**	Ex 10	1/4
	Nonsense	c.833T>G	p.L278*	Ex 8	2/4
	Deletion	**c.1096_1104del**	p.V366*	Ex 10	1/4
*SLC45A2*	Missense	c.328G>A	p.G110R	Ex 1	1/4
		c.478G>C	p.D160H	Ex 2	3/4

Note

Novel mutations are in bold.

Three mutation alleles were identified in two patients who were affected by OCA type 2. One of them (Patient 15 in Table 1) was heterozygous for two novel mutations that included one frameshift mutation (c.1096_1104del, P.V366*) and one missense mutation (c.1079C>T, p.S360F) (Figure 2b,c). Another one was homozygous for nonsense mutation (c.833T>G, p.L278*) which had been reported previously. (Table 4).

We also identified two mutation alleles in two patients affected by *SLC45A2*. One patient was compound heterozygous for c.328G>A (p.G110R) in Exon 1 and c.478G>C (p.D160H) in Exon 2. The other was homozygous for c.478G>C (p.D160H). The mutations were missense mutation and had been previously reported.

### 3.2 Prenatal genetic diagnosis

Four high‐risk fetuses were screened for *TYR* mutation according to their parental genotypes. Three of them did not carry any detectable mutation and none of these three subjects showed symptoms of OCA after birth. One fetus carried heterozygous mutant *TYR* alleles (Table 3). However, this fetus was spontaneously aborted for uncertain reason at the ninth week of gestation.

**Table 3:** Prenatal diagnosis and outcome of four high‐risk fetuses

Predigree	Molecular Subtype	Involved gene	Proband genotype		Fetus genotype	Fetus outcome	Baby phenotype
			Paternal allele	Maternal allele			
1	OCA1	*TYR*	c.832C>T(p.R278*)	c.1199G>T(p.W400L)	No mutation	Normal birth	Normal
2	OCA1	*TYR*	c.1204C>T(p.R402*)	c.71G>A(p.C24Y)	No mutation	Normal birth	Normal
3	OCA1	*TYR*	c.832C>T(p.R278*)	c.346C>T(p.R116*)	No mutation	Normal birth	Normal
4	OCA1	*TYR*	c.1199G>T(p.W400L)	c.832C>T(p.R278*)	c.832C>T(p.R278*)	Spontaneous abortion	─

Note

A dash (─) indicates no relevant information.

### 3.3 In silico analysis and structural simulation of novel mutations

**Figure 3:**
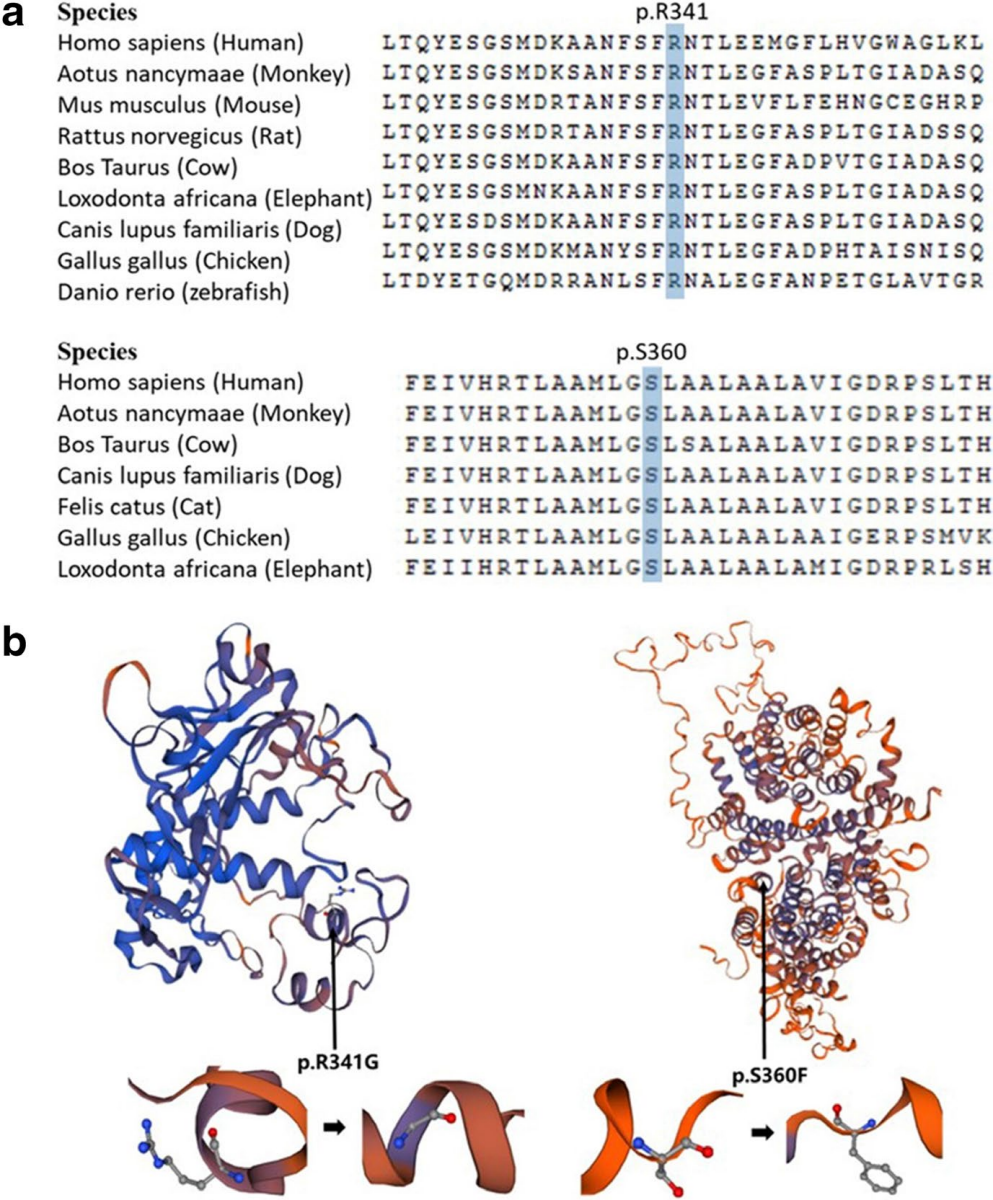
(a) The R341 in TYR and S360 in P protein are highly conserved amino acids throughout evolution; (b) Simulation of the amino acids conformation changes by SISWS‐MODEL

The three novel mutations, c.1021A>G (p.R341G), c.1079C>T (p.S360F), and c.1096_1104del (p.V366*), were all predicted as disease‐causing mutations by Mutation Taster, Polyphen and SIFT (Table 4). None of them was present in Albinism Databases, the 1,000 Genomes Database, gnomAD database, or the HGMD Professional Database. The R341 in TYR and S360 in P protein are highly conserved amino acids throughout evolution (Figure 3a). The R341G was assessed to decrease the TYR protein stability while S360F was predicted to increase the P protein stability by I‐Mutant2.0. Significant conformation changes of amino acid and polypeptide caused by the mutations can be observed through the simulation of SISWS‐MODEL (Figure 3b).

**Table 4:** Bioinformatic analysis results of the three novel mutations

Novel mutation	Bioinformatic analysis					
	Mutation Taster		Polyphen 2		SIFT	
	Prediction	Score	Prediction	Score	Prediction	Score
p.R341G	Disease causing	0.999	Probably damaging	1.000	Affect protein function	0.01
p.S360F	Disease causing	0.999	Probably damaging	0.998	Affect protein function	0.00
c.1096_1104del	Disease causing	1				

### 3.4 Molecular pathology evaluation

According to the standards and guidelines for interpretation of sequence variants developed by the ACGM (Richards et al., 2015). Both of the c.1021A>G (p.R341G) and c.1079C>T, p.S360F mutations were evaluated as a pathogenic variant with one strong (PS2), two moderate (PM1, PM2), and four supporting (PP1, PP2, PP3, PP4) pathogenicity evidences. The pathogenicity of the deletion variant, c.1096_1104del (p.V366*) was determined as pathogenic by one very strong (PVS1) and one strong (PS2) pathogenicity evidences.

## 4 DISCUSSION

In this study, we performed mutation analysis of the *TYR*, *OCA2,* and *SLC45A2* on 18 unrelated OCA patients and *TYR* analysis on four fetuses at risk for OCA. Mutations in *TYR* were the most frequent (77.8%) in this tested group, whereas mutations in *OCA2* (11.1%) and *SLC45A2* (11.1%) occurred more rarely than previously reported (Kamaraj et al., 2014; Khordadpoor‐Deilamani, Akbari, Karimipoor, & Javadi, 2015).

A total of 28 OCA type 1 mutational alleles were found in this report (Table 2), including c.929_930insC in eight alleles, c.832C>T in five alleles, c.896G>A in four alleles, c.1199G>T and c.346C>T in two alleles, c.1021A>G, c.1106A>G, c.636A>T, c.71G>A, c.895C>T in one allele, respectively. Missense mutation (46.4%, 13/28) in our study was the most common mutation in OCA type 1 patients, which is consistent with the previous study (Ghodsinejad et al., 2014). The insertion mutation, c.929_930insC, located on *TYR* exon 2, was the most common (28.6%, 8/28) *TYR* mutation (Table 2) in this study. This mutation has been reported to prevalent in Chinese OCA type 1 patients (Wang, Guo, Li, & Lian, 2009; Wei et al., 2010) and other east Asian countries such as Japanese and Korean (Goto et al., 2004; Park, Chae, Kim, & Kim, 2012; Suzuki & Tomita, 2008). The c.929_930insC frameshift results in a truncated tyrosinase polypeptide lacking one potential copper‐binding region which will lead to completely lack of tyrosinase catalytic activity (Tomita, Takeda, Okinaga, Tagami, & Shibahara, 1989).

Another common *TYR* mutation in our study was c.832C>T(p.R278*) (17.9%, 5/28). This nonsense mutation results in the deamination of 5‐methylaytosine at a CpG site (Tripathi et al., 1993) and the enzyme may completely inactivated for lacking one potential copper‐binding region (Renugadevi, Sil, Perumalsamy, & Sundaresan, 2010). It was first reported in Indo‐Pakistani patients (Tripathi et al., 1993) and then reported in various ethnic groups, such as European (King et al., 2003), Mexican(King et al., 2003), Jewish (Gershoni‐Baruch et al., 1994; Goto et al., 2004), Indian (King et al., 2003), Japanese (Goto et al., 2004; Tomita & Miyamura, 1998), and Chinese (Wang et al., 2009). The frequency of the mutation was 17.9% in our study, which corresponds with a previous study in Chinese (18.8%) (Wang et al., 2009).

The third common mutation in *TYR* in our study was c.896G>A (p.R299H) (14.3%, 4/28) which is missense substitution in the central portion of tyrosinase. R299H missense mutation occurs within the so‐called “CuA” region, which might be important for binding of atomic copper by these proteins. This mutation was first reported in Caucasian by Tripathi, Strunk, Giebel, Weleber, and Spritz (1992) and R299H substitution has been observed previously in Caucasian (Tripathi et al., 1992), Arab Christian (Gershoni‐Baruch et al., 1994), Korean (Lee, Park, Lee, Lee, & Park, 1997), and Chinese (Wang et al., 2009) populations.

The two *TYR* mutations in our study, p.W400L and p.C24Y, have only been reported in Chinese OCA patients (Lin, Chien, Su, Lee, & Chen, 2006; Tsai et al., 1999; Wang et al., 2009), while p.R116*, p.Y369C, p.R212S, and p.R299C have also been described in Caucasian patients. Patient 8 was compound heterozygous for a known mutation (c.929_930insC, p.R311Lfs*7) and a novel mutation (c.1021A>G, p.R341G) in *TYR*. The p.R341G located in the copper B binding site of tyrosinase and might affect the protein function by impeding atomic copper binding.

Patient 15 was heterozygous for two novel mutations (c.1096_1104del, p.V366* and c.1079C>T, p.S360F) in *OCA2*. Mutation c.1079C>T (p.S360F) lead to a substitution of serine to phenylalanine at the middle of the third transmembrane domain of P protein, where a small perturbation can lead to inactivity of the P protein. Mutation c.1096_1104del located at the end of the third transmembrane domain and resulted in a truncated protein. This frameshift would be expected to result in a loss of function and might lead to inactivity of the P protein (Toyofuku et al., 2002).

Interestingly, Patient 15 was a 3‐month‐old girl with OCA type 2 who had a solitary pigmented nevus with a diameter of 3 mm in the right inguinal area. For the right inguinal area was a sun‐protected area, the pigmented nevus was not a sun‐related lesion. The genetic defect for OCA type 2 has been mapped to chromosome 15q11‐q13. Individuals with OCA2 possess some degree of tyrosinase activity and therefore can produce melanin. This may explain the pigmented nevus that usually happens in OCA type 2 patients in this and other similar studies (Stevens, Ramsay, & Jenkins, 1997; van der Westhuizen, Beukes, Green, Sinclair, & Goedhals, 2015).

Moreover, we genotyped for four high‐risk fetuses for mutations in *TYR*. The fetus which was spontaneous abortion at ninth week carried a single mutation allele of c.832C>T (p.R278*). The other three fetuses carried no *TYR* mutations, and showed no signs of OCA type 1 after birth. These data confirmed that prenatal genetic testing is possible for OCA disease prevention.

In conclusion, a total of 15 mutations were found in 18 Chinese OCA patients and four high‐risk fetuses. Three novel mutations, c.1021A>G mutation in *TYR,* and the c.1096_1104del and c.1079C>T mutations in the *OCA2,* were first reported. All the novel mutations were assessed to be responsible for affecting the protein function and predicted to cause OCA. What's more, the successful prenatal genetic tests for OCA helped to find out the reason of spontaneous abortion and will make a significant sense to reduce the burdens of the family and the society. These outcomes will expand the genotypic spectrum of OCA and highlight the importance of genetic counseling for families with OCA history or abortion history, and pave the way for more efficient carrier detection and genetic counseling.

## CONFLICT OF INTEREST

None declared.
