# Supplementary material for: Mutational Analysis of TYR, OCA2, and SLC45A2 Genes in Chinese Families with Oculocutaneous Albinism
Source: Mol Genet Genomic Med. 2019 Jun 14;7(7):e00687. doi: 10.1002/mgg3.687 (PMC6625147; doi:10.1002/mgg3.687)
Supplement: Supplementary file 1 [file MGG3-7-e00687-s001.docx]

Supplementary Table 1: Primers of 5 exons of *TYR* gene

| Exon | Forward | Reverse | Size（bp） |
| --- | --- | --- | --- |
| 1-1 | CCCACTGGTGGGATA GA | GGTCCCCAAAAGCCA AC | 553 |
| 1-2 | CCCTAGAGCCTGTGTCTC C | CCCTGCCTGAAGAAGGAT | 827 |
| 2 | CCTCAGGAGAAGTCTAC AAC | ACAACACATATTCTTGGTC | 446 |
| 3 | TGGGTATCCAGAATGTAA A | TTTAAATCCAATGAGCACG | 356 |
| 4 | TTTTAATATATGCCTTAT TTT AC | GGTAACACTAGATTCAGC AA | 321 |
| 5 | CTCCAAAGGACTGTGAAA GGA | GGTCTTTACAGAAAAATA C | 415 |

**GenBank reference sequence and version number for *TYR*: NC_000011.10**

Supplementary Table 2: Primers of 25 exons of *OCA2* gene

| Exon | Forward | Reverse | Size（bp） |
| --- | --- | --- | --- |
| 1 | GAGTTCTTACTTCGA | TAAACCCTCCCTGCCTGTTC | 172 |
| 2 | GGTGCAAACGTTAGTCTCAG | CCAATCTGTGTGAAGTCCAC | 359 |
| 3 | CTGGGAACACATACATTATT | GTGCAATGCTCAGAAACTCT | 209 |
| 4 | AAGCTTGCTTTGTAGCCATT | CGATGTGCGGCCACCGCTGC | 300 |
| 5 | AGTGTCTGAGTCTGGGCAA | CAGAGAATCAGGCGAAGAG | 177 |
| 6 | ATTTATACCTTACTGCTCTC | TTTCAGATCTCAGCCAGGCG | 183 |
| 7 | GGACATGGGGTTTCTCCTGT | TGAGATGAAATGAGATTTCAC | 264 |
| 8 | AGATCCCAGATGGTGTCTCA | AGGTCAGACTCCTTTAAACG | 213 |
| 9 | AGAGGGAGGTCCCCTAACTG | ATCTCAAGCCTCCCTGACTG | 272 |
| 10 | CTTTCGTGTGTGCTAACTCC | ACATCTTTGAGCTGACATCC | 195 |
| 11 | GCAGCGCTTCATTAGGCTCA | GGCCAGAGAAGGCCCGGTTA | 201 |
| 12 | GTCGTTTTAATATGGTGGCC | CCTGCAGAAGCAACCTTTA | 256 |
| 13 | GCCTCTGTTCTACGAGCCTG | TGCCAGAACCTGGCCGCAA | 231 |
| 14 | TTCACGATGTGTATAGTGGG | AAGTGGAGGTGTGCGTTTAC | 239 |
| 15 | GATTACAGGCGTGAGCCACC | ACCCATCAACAGATACTTCC | 293 |
| 16 | GAGGGTGTTGCTGATATCTG | GAATGTTCTGCTGCACACCA | 260 |
| 17 | AGGCTCCAAGTCACAGACCG | CTTCTTGGAGAAGTGAATCAG | 219 |
| 18 | AGTTGCGTAGGTTATGACAC | CCCATCCAGAATGTGACAAA | 235 |
| 19 | GTTATGTATTTGCAGCCCCT | AATCCACCAAATACAATTGA | 197 |
| 20 | GAATCGGTGTGTTAACAGTG | GTAGGCTTTCTTCATTCACC | 266 |
| 21 | GCCTACCTTATGTTCACGTC | AATCAAAGAACAGTGGCTGG | 212 |
| 22 | TGGTGGGTCTGACCCTAAGT | AGGCTATGTCCAGGCTAAAG | 219 |
| 23 | ACAGTATGGCAGCTTCTCTG | ACTAACTGTTGCTTTGGGCT | 229 |
| 24 | GAGAACAGAAGCTTACCACC | GCTTAGGAACTAGACAGTTTA | 204 |
| 25 | CGTATCTCATGAGCTTATCC | AGCATACAATTTGAATGCTG | 574 |

**GenBank reference sequence and version number for *OCA2*: NC_000015.10**

Supplementary Table 3: Primers of 7 exons of *SLC45A2* gene

| Exon | Forward | Reverse | Size（bp） |
| --- | --- | --- | --- |
| 1-1 | AGGCTCCACGTCAAATCCAG | GGTCACATACGCTGCCTCCA | 260 |
| 1-2 | CAGACTCATCATGCACAGCA | ATGCCCACGAGCATCATGAC | 252 |
| 1-3 | CAGCATTGTGTGGTTCCTCA | GGTCAAACACATGAACATCCTC | 261 |
| 2 | AACGTGGATGATTCTAAAACAGGA | CTCATTGTCTGGGGAGCTGA | 280 |
| 3-1 | GGGAGTGTCTATGCATGAGG | GATAGAACCATACTCGTACATTCC | 324 |
| 3-2 | GCCCCACTTACAGAGGTTGC | CAACAAAGAGCAAGAATATTTTCCCTTG | 224 |
| 4 | AGCTGGCTGAGTTTCTGCAG | CCTCAACAGGTGTTAATGGAGG | 265 |
| 5 | AGAGGTGGAGAAGCAGAGTG | GAAGACATCCTTAGGAGAGAG | 236 |
| 6 | ATGAGGCACTGCCAGCTGTA | CCCAAGGCAGAGGTTCAATG | 286 |
| 7 | GCCCTAAATGACAGTTCCTTG | TGTGCTTCACTGTCTCTGAG | 326 |

**GenBank reference sequence and version number for *SLC45A2*: NC_000005.10**
